# On the Treatment of Wounds and Ulcers, and Hæmorrhage

**Published:** 1815-06

**Authors:** Richard Walker

**Affiliations:** of Oxford.


*For the Medical and Physical Journal.*

### *On the Treatment of Wounds and Ulcers, and Hæmorrhage;* by Mr. RICHARD WALKER, of Oxford.

*(Continued from p. 230.)*

THE propriety, as I have inculcated, of dressing certain ulcers and sores every morning at an early hour, and every evening, viz. at about equal distances of time in the twenty-four hours, is an extremely inconvenient circumstance to the practitioner; but the comfort and utility of it to the patient is incalculably great, and the necessity of it, in serious cases, indispensible.

The application of dry lint to sores is much more important than is ordinarily apprehended, and not so obvious in the mode of management as is commonly conceived. In addition to the ordinary intention, and mode of using it, I shall observe, 1st, that it is necessary as an absorbent of matter, matter, by which it likewise tends to cleanse, and keeps sores in a clean and healthy state; 2dly, it keeps down exuberant flesh or granulations; and 3dly, it acts as a desiccative and cicatrizer. In this latter intention, used according to the following method, it supersedes in utility the various absorbent powders, commonly used for this purpose, viz. lapis calaminaris, creta, &c. particularly in sores of large surface, as in burns, scalds, excoriations, &c.

I apply dry lint to sores from the beginning to the end of the cure, or at least so long as there is any discharge, under the plaster, whether it be adhesive plaster or cerate, adjusting the quantity so as it shall be just sufficient to absorb the discharge, of course diminishing it gradually, till at last, as in small sores, I use the mere down of the lint; and in all cases (except where pressure is required, as in redundancy of flesh, or to give firmness to the part) I lay it on as loosely and as lightly as possible, so that the plaster or cerate may apply, as it were, between or through the interstices of the lint; and when such sore is small, and very nearly healed, I apply the lint beyond or somewhat over the edges of the sore, observing, however, that it be so thin, as that the dressing of cerate over shall bring it clean off, without sticking to or tearing the edges. By this means, and a moderately tight roller, sores may be healed more firmly and naturally than by the application of ordinary cicatrizers, so called; and this method I prefer, where it is efficient, to the ordinary escharotic applications, as the nitrate of silver, sulphate of copper, &c. in cases of exuberant granulations, conceiving this a much more natural way, and the cure, in consequence, more firm and perfect.

In the extensive superficial sores which succeed to burns or scalds, and likewise in excoriations which discharge a thin acrimonious fluid, I strew them over lightly, after the manner above-mentioned, with the down of lint, and apply a dressing of drying cerate spread on fine soft old linen.*

It is necessary to remark here, that, under certain circumstances, the application of dry lint, under plaster or cerate, is injurious, viz. when it is not thrown or brought off by the dressing. This happens in certain dry or crude sores, and is frequently the case in persons whose occupation requires them to stand or walk much during the cure.

In all instances of recent wounds, it is of importance, for expedition sake and, to prevent a scar, to keep in view the

---

* The drying cerate I use, is the one recommended by me in the same intention, in burns and scalds; and linen, in such cases, is much preferable to lint.

prospect, prospect of healing by the first intention, as it is called: therefore, in all instances where the hæmorrhage, though troublesome, proceeds only from small vessels, the lips of the wound should be brought into close contact, and the part encircled or surrounded, if it can be, as in the wrist, fingers, &c. by strips of adhesive plaster, wrapping well over each other, and afterwards secured by moderately tight bandage.

My method, in such cases, is to place a line of lint, alone, or covered with flour, in the direction of the wound, as a kind of check to the hæmorrhage, previously to applying the plaster, &c.

If, however, it be necessary, in order to stop the hæmorrhage, to introduce agaric, or floured lint, within the wound, the gap thus made should, as in every other instance of wounds or ulcers, be afterwards brought progressively together, as much as possible, by the due application of adhesive plaster and bandage at every subsequent dressing.

With respect to the fittest kind of adhesive plaster, as an application to sores and ulcers, such should be chosen (if not prepared by the surgeon himself) as is not so brittle as to scale off from the cloth, and which is so spread as to give a thickish or strongish coating to the cloth, and of a slight yellowish shining complexion. The adhesive plaster, procured from different manufacturers of this article, varies much respecting these particulars.

The ordinary progress of an ulcer or sore, except in the instances of certain fresh wounds, which are sometimes healed by the first intention, as it is called, may be divided into three stages, viz. digestion, incarnation, and cicatrization; the rules for effecting the former of these are comprehended in the foregoing directions.

With respect to incarnation, I believe I may venture to affirm, that there is no article known which possesses the quality, specifically, of generating new flesh in any wound or sore: this process, however, naturally takes place in every sore which is preserved in a perfectly clean and healthful state, and is, therefore, the necessary consequence of a judicious perseverance in the mode of treatment for digestion, &c. before described.*

The cicatrization of sores, in my opinion, should be effected, if possible, by the judicious application of dry lint, proportioned to the dimensions of the sore, laid lightly on,

* Certain preparations of mercury, as the nitric oxyde of mercury, &c. act by producing a fresh clean surface on a sore, and thus promote, in a peculiar degree, the generation of new and healthful flesh.

using in small sores the down of the lint only, diminishing the quantity according to the contraction of the sore, applying over the lint the ordinary plaster, or, which is sometimes better, a drying goulard cerate of hardish consistence, with proper bandage dextrously applied with due pressure, which very much facilitates this intention.

I have found, especially if adhesive plaster be used, that the thin stratum of lint is applied with best effect, for skinning over the edges of a sore, by being extended somewhat beyond the edges of the sore, thus acting as a desiccative, and consequently as a cicatrizer.

By this method, sores, I am convinced, are healed more firmly and uniformly, wherever pressure can be made, than by the artificial means of cicatrizers, so called, of any description. Moreover, any exuberant granulations may, ordinarily, be prevented from rising, or be removed by this means, and, consequently, much unnecessary pain spared to the patient, incident to the application of escharotics.

If, however, cicatrizers or escharotics are found indispensibly necessary for either of these purposes, the argentum nitratum, or cupri sulphas, may be used either in substance or in solution.

Having long observed that cicatrization by either of the above articles, especially if used in the solid state, is not of so firm or natural a state as by the means I have recommended, I carefully avoid (should I have occasion to touch the central or inner parts of a sore) applying it, especially if it be the cupri sulphas, to the edges of the sore.

In large fungus sores, of course, even the lapis infernalis must occasionally be resorted to. Argentum nitratum possesses the property as a coalescent as well as a cicatrizer, to such an extraordinary degree, that by the skilful application of it, either in its solid state, or in solution, according to circumstances, assisted by compression with bandage, the detached parts occurring in ulcers of every description almost, particularly scrofulous or venereal, may be readily made to coalesce and heal firmly, as I have ordinarily experienced; and, in some instances, even sinuses, by injection and pressure.

Small sores, which have resisted long the ordinary means, are sometimes healed at once, or disposed to it, by a touch of the argentum nitratum, and sometimes by the ceratum hydrarg. nitrat. rub. The former is indicated in an inactive or sluggish state of the sore; and the other when foul, or covered by thin sordes, which adhesive plaster may have failed in removing.

I consider the introduction of adhesive plaster into general ral practice as an application to ulcers and sores, according to the plan of Mr. Baynton, as a discovery, for so I presume I may call it, of the first importance, for utility, in surgery.

Whoever has experienced the manner of treating sores and ulcers, previous to the introduction of Mr. Baynton's method, which is of late date only, and appreciates duly the value of the present improved method, comparatively with the former, must, with myself, consider it as a truly great acquisition.

It happens, however, that persons who have entered into the profession since the introduction of this improved method, adhere to it, as I have witnessed, too implicitly, and are, moreover, at a loss how to proceed when a deviation from this mode becomes necessary.

The use of adhesive plaster admits almost of a general application, with respect to utility; but there are, nevertheless, certain exceptions to its use, which I shall endeavour briefly to point out.

The beneficial effects of this application, independantly of, or in addition to, its well known use in retaining or approximating the lips or edges of wounds and ulcers together, depends, as I consider it, on the following principles:—1st, in excluding, effectually, the external air from such sore; 2dly, by keeping the pores open, that is, occasioning, ordinarily, a sensible exudation from the surrounding skin; and 3dly, by cleansing and promoting, specifically, the digestion of such sore: which effects are not so well produced, in ordinary sores or ulcers, by any cerate or unguent.

From this brief statement of the means by which adhesive plaster acts upon ordinary sores and ulcers, it will be apparent where its use should be adopted and where rejected, viz. in all instances where considerable excoriation or intense inflammation occurs, and in certain irritable sores, unless in such instances it produces a salutary discharge, which operates in removing or diminishing such effects, it should be forborne, till these have subsided, and other means, as anodyne and emollient fomentations and poultices, adopted.

Much also depends upon the composition and consistence of the plaster. I am not prepared to give what I consider the fittest formula for this purpose; neither, indeed, can any precise formula be given, to suit, equally, every variety of circumstances respecting the state of the sore and the patient. In general, however, it should be moderately, or rather strongly adhesive; of such a consistence and composition as to be hard and tenacious, without being brittle, or liable to scale off from the cloth; and lastly, it should have have a fair or strong face upon the cloth on which it is spread, and, when taken off, should leave none of the plaster behind adhering to the skin.

It must not be forgotten, that even in some instances where the application of an emollient or drying cerate is more adapted to the sore itself, that a covering of adhesive plaster may be serviceable, for the purpose of drawing or approximating the skin and lips of the sore towards contact.

In all cases where the object is to approximate or bring together the edges of a wound or ulcer, of course the greatest purchase for that purpose should be made; this is best effected, where the part will admit of it, by surrounding the limb with the plaster in the manner mentioned when speaking of its application in hæmorrhage.*

N.B.—In a certain dry or harsh state of the skin, accompanying sores or excoriations, I have found that goulard liquor, used either for a poultice or a lotion, is not so proper an application as milk, milk and water, or water alone, used after the same manner. Likewise, where an exudation from the skin is desirable, certain leaves possessing a kind of extractive relaxant property, as the young leaves of the colewort or cabbage-plant, prepared in the usual manner, and applied over a dressing immediately covering the sore, is occasionally serviceable.

With respect to the efficacy of an issue, as a substitute for habitual sores or ulcers, and the mode of managing it, I shall have occasion to speak of hereafter.

With regard to any medicines which might be necessary in sores or ulcers, not depending upon, or connected with, any specific constitutional affection of the system, cooling aperients, with an antiphlogistic regimen; or moderate doses of calomel, as an alterative; or the bark, as a tonic; and opium, as a sedative or anodyne; each, according to circumstances, will, in ordinary cases, be all that may be required; recollecting, as a restorative or corroborant, never to forget the most efficacious of all means, viz. due regimen, and a moderate administration of cordials, of which good port wine or Madeira are the best.

It is at this time the fashion to administer the bark in port wine, thus combining in one medicine, where a cordial and tonic are both judged necessary, the respective powers

* In order to prevent tumefaction or tension coming on, or to remove it, if present, which is frequently the case in fresh wounds, the same means may be resorted to, respecting the application of cold, &c. as is mentioned in the treatment for hæmorrhage.

 of of each. I am of opinion, however, that the proper time for administering bark in such cases, whether in substance or decoction, is at due distances between meals; but the port wine, as a tonic cordial, never, unless under certain temporary urgent circumstances, upon an empty stomach. It may be proper to join some aromatic with the bark, as confectio aromatica or pulvis zingiberis.

*Oxford.* RICHARD WALKER.

*(To be continued.)*

